# Neuronal-driven glioma growth requires Gαi1 and Gαi3

**DOI:** 10.7150/thno.61452

**Published:** 2021-07-25

**Authors:** Yin Wang, Yuan-yuan Liu, Min-bin Chen, Kai-Wen Cheng, Li-na Qi, Zhi-qing Zhang, Ya Peng, Ke-ran Li, Fang Liu, Gang Chen, Cong Cao

**Affiliations:** 1 Jiangsu Key Laboratory of Neuropsychiatric Diseases and Institute of Neuroscience, Soochow University, Suzhou, China.; 2 Clinical research & lab center, Affiliated Kunshan Hospital of Jiangsu University, Kunshan, China.; 3 Department of Radiotherapy and Oncology, Affiliated Kunshan Hospital of Jiangsu University, Kunshan, China.; 4 Department of Neurosurgery, The Third Affiliated Hospital of Soochow University, Changzhou, China.; 5 The Fourth School of Clinical Medicine, The Affiliated Eye Hospital, Nanjing Medical University, Nanjing, China.; 6 Department of Neurosurgery, Nanjing Medical University Affiliated Changzhou NO.2 People's Hospital, Changzhou, China.; 7 Department of Neurosurgery, the First Affiliated Hospital of Soochow University, Suzhou, China.; 8 The Central Lab, North District, The Affiliated Suzhou Hospital of Nanjing Medical University, Suzhou Municipal Hospital, Suzhou, China.

**Keywords:** Neuron-glioma communication, NLGN3, Gαi1/3, Signaling

## Abstract

Neuroligin-3 (NLGN3) is necessary and sufficient to promote glioma cell growth. The recruitment of Gαi1/3 to the ligand-activated receptor tyrosine kinases (RTKs) is essential for mediating oncogenic signaling.

**Methods:** Various genetic strategies were utilized to examine the requirement of Gαi1/3 in NLGN3-driven glioma cell growth.

**Results:** NLGN3-induced Akt-mTORC1 and Erk activation was inhibited by decreasing Gαi1/3 expression. In contrast ectopic Gαi1/3 overexpression enhanced NLGN3-induced signaling. In glioma cells, NLGN3-induced cell growth, proliferation and migration were attenuated by Gαi1/3 depletion with shRNA, but facilitated with Gαi1/3 overexpression. Significantly, Gαi1/3 silencing inhibited orthotopic growth of patient-derived glioma xenografts in mouse brain, whereas forced Gαi1/3-overexpression in primary glioma xenografts significantly enhanced growth. The growth of brain-metastatic human lung cancer cells in mouse brain was largely inhibited with Gαi1/3 silencing. It was however expedited with ectopic Gαi1/3 overexpression. In human glioma Gαi3 upregulation was detected, correlating with poor prognosis.

**Conclusion:** Gαi1/3 mediation of NLGN3-induced signaling is essential for neuronal-driven glioma growth*.*

## Introduction

Glioma is the most common type of brain tumor of either astrocytic or oligodendroglial origin 1, 2. High-grade gliomas, such as glioblastoma (GBM) and anaplastic astrocytoma, cause human mortality both in children and adults 3, 4. Dysregulation of multiple signaling cascades and complex signaling crosstalk are vital in the tumorigenesis and progression of human glioma 5-9. These cascades include phosphoinositide 3-kinase (PI3K)-Akt-mammalian target of rapamycin (mTOR) 10, p53, retinoblastoma (Rb), epidermal growth factor receptor (EGFR) and Wnt/β-catenin 11 as well as signal transducer and activator of transcription (STAT), neurotrophic tyrosine receptor kinase (NTRK), fibroblast growth factor receptor (FGFR) and many others 5, 6. It is therefore extremely important to identify the dominant and key driving signaling molecules of glioma in order to develop efficient targeted therapies 5, 6, 12, 13.

Recent studies have revealed that neuronal activity and neuron-glioma circuits are vital for the malignancy of human glioma, and that the neuronal activity could be a key determinant for glioma tumorigenesis and progression 14-16. Studies highlighted a crucial role of neuroligin-3 (NLGN3) in the mechanism of glioma progression 17-20. NLGN3, a synaptic protein cleaved and secreted in an activity-dependent manner, was identified as the primary factor responsible for glioma progression 17-20. Xenografts derived from high-grade primary human glioma cells failed to grow in a NLGN3-deficient brain 18, demonstrating that NLGN3 is required for glioma cell growth. Furthermore, inhibition of ADAM10 (A Disintegrin And Metalloproteinase 10) blocked activity-dependent NLGN3 secretion to inhibit orthotopic growth of primary glioma xenografts 19.

NLGN3 secreted from neurons was found to induce phosphorylation and activation of several key receptor tyrosine kinases (RTKs) on glioma cells to promote glioma progression 17-19. Additionally, NLGN3-activated glioma cells produce feedforward expression of NLGN3 to further increase glioma cell growth and proliferation 17-19. Recent studies found that NLGN3 induces the expression of synaptic genes required for neuron-glioma synapse formation, as well as genes essential for glioma proliferation and invasiveness 14, 15. These studies identify NLGN3 as a key neuronal-derived factor for promoting activity-dependent glioma growth 17-19.

Gαi proteins, the inhibitory α subunits of G proteins (heterotrimeric guanine nucleotide-binding proteins), have three primary family subunits, Gαi1, Gαi2 and Gαi3 21. Through binding to GPCRs (G protein-coupled receptors) Gαi proteins and the βγ complexes can suppress adenylate cyclase (AC) to deplete cyclic AMP (cAMP) levels 21. Gαi proteins are inhibited by pertussis toxin. Glider *et al.,* showed that pertussis toxin potently inhibited migration and invasion of high grade glioma cells 22. In addition, pertussis toxin and temozolomide co-treatment resulted in better anti-glioma efficiency in a rat intracerebral glioma model 23. Moreover, a D2 dopamine receptor agonist stimulated mitogenesis of C6 glioma cells through activation of pertussis toxin-sensitive Gαi proteins 24.

Alternatively, our studies show that Gαi1 and Gαi3 proteins also play a key role in mediating the signal transduction of several RTKs, including EGFR (epidermal growth factor receptor) 25, fibroblast growth factor receptor (FGFR) 26, keratinocyte growth factor receptor (KGFR) 27, brain derived neurotrophic factor (BDNF) receptor TrkB 28, and vascular endothelial growth factor receptor 2 (VEGFR2) 29. Specifically, the recruitment of either Gαi1 or Gαi3 subunit to these ligand-activated RTKs was found to be essential for mediating PI3K-Akt-mTORC1 and Erk-MAPK signaling 25-29. As neuron-secreted NLGN3 activates RTKs to promote glioma progression, we explored the potential function of Gαi1 and Gαi3 in NLGN3-induced signaling and glioma cell progression.

## Methods

**Ethics.** Protocols of this study were approved by the Ethics Committee of Soochow University.

**Reagents.** From Gibco (Shanghai, China) cell culture reagents were obtained. The antibodies were described early 26. NLGN3, pertussis toxin (PTX), LY294002 and PD98059 were purchased from Sigma-Aldrich (St. Louis, Mo). All primers, sequences and viral constructs were provided by Shanghai Genechem Co. (Shanghai, China), unless otherwise mentioned.

**Cell lines.** U251MG glioma cell line was obtained from the Cell Bank of Shanghai Biological Institution (Shanghai, China), cultivated as described. The cell line has previously been tested and authenticated by the Cell Bank of Shanghai Biological Institution. Wild-type (WT), Gαi1 and Gαi3 double knockout (DKO), Gαi1, Gαi2 or Gαi3 single knockout (SKO) mouse embryonic fibroblasts (MEFs), as well as WT and Gab1 KO MEFs, were cultivated as previously described 25, 27-30. Cells were starved in 0.5% FBS medium overnight plus 30 min warm PBS for signaling analyses.

**Human glioma tissues.** According to principles of the Declaration of Helsinki, this study was approved by the Ethics Committee of Soochow University. Human tissues, including the glioma tissues and surrounding normal brain tissues, were described previously 26. Tissues were obtained from The First, Second, Third Affiliated and Children Hospitals of Soochow University. All participates provided written-informed consent.

**Primary culture of human cancer cells.** The source and culturing of primary human glioma cells were previously described 26. From a lung cancer patient with brain metastases (female, 50 years old, brain metastatic lung papillary adenocarcinoma, CK7^+^, CK20^—^, Villin^—^, TTF-1^+^, CDX2^—^, PAX8^—^, CD10^—^, CAIX^—^, PR^—^, EMA^+^, GFAP^—^), surgical removed brain metastatic cancer tissues were washed, minced into small pieces, and digested with Collagenase I and DNase (Sigma-Aldrich). Single-cell suspensions were then pelleted and washed. Fibroblasts, blood vessel cells, immune cells and other non-cancerous cells were abandoned. Purified brain-metastatic human lung cancer cells (bmLCs) were *ex vivo* cultured in the described medium 31. The study protocols were reviewed and approved by the Ethics Committee of Soochow University, and conformed to the guidelines of Helsinki declaration. The informed consent was obtained from all subjects before their participation.

**Quantitative real-time reverse transcriptase polymerase chain reaction (qPCR).** As described, qPCR was carried out by an ABI 7600 Prism equipment through utilizing a SYBR Green PCR kit. *Gαi3* and *NLGN3* mRNA levels were quantified by a ^ΔΔ^Ct protocol 32, using *GAPDH* as the internal control 26. Primers are listed in **Table 1**.

**Western blotting and co-immunoprecipitation**. Protocols for Western blotting and co-immunoprecipitation (co-IP), as well as data quantification, have been extensively described previously 25, 28. For all Western blotting assays, each lane was loaded with exact same amount of quantified protein lysates (30-40 μg in each treatment). Same set of lysate samples were run in parallel (“sister”) gels to test different proteins when necessary. Expression of indicated proteins was quantified via ImageJ software, with results normalized to the equal loadings.

**Endosome fractions.** Cells with the applied treatments were harvested and re-suspended in the hypotonic swelling buffer 33, and lysed with 30 strokes in a Dounce homogenizer using a tight pestle, and swelling was stopped by the addition of two fold homogenization buffer 33. Lysates were centrifuged to obtain the post-nuclear supernatants, and centrifuged 33. The resulting supernatants were centrifuged, and the pellet solubilized in the homogenization buffer 33. Insoluble particles were removed by short centrifugation and the supernatant loaded onto a 5-20% continuous OptiprepTM (Sigma-Aldrich), poured using homogenization buffer. The gradient was further centrifuged at 60,000 g for 24h, with total 10 endosomal fractions collected, and proteins precipitated with 12% TCA for 1h. Fractions were centrifuged at 12,000 g for 1h. The protein pellets, combining all ten endosomal fractions, were dissolved in SDS-sample buffer for analysis by Western blotting.

**Gαi1/3 shRNA.** Glioma cells or bmLCs were seeded into six-well plates at 50-60% confluence, treated with Gαi1 shRNA lentiviral particles (sc-105382-V) (Santa Cruz, CA) and Gαi3 shRNA lentiviral particles 29. After 24h, cells were further cultured in puromycin (1.0 μg/mL)-containing complete medium for 12-14 days. Gαi1/3 knockdown (over 95% efficiency) in stable cells was confirmed by Western blotting. shRNA-mediated knockdown of Gαi1 and Gαi3 in MEFs was reported previously 27-29. Gab1 shRNA lentiviral particles, for both human and mouse, were also obtained from Santa Cruz Biotech.

**CRISPR/Cas9 knockout of Gαi1 and Gαi3.** The lentiviral CRISPR/Cas-9 Gαi1 KO construct and the lentiviral CRISPR/Cas-9 Gαi3 KO construct were provided by Shanghai Genechem (Shanghai, China), transfected into MEFs, and selected with puromycin. Gαi1/3 knockout was confirmed by Western blotting. Control cells were treated with the empty vector with nonsense sgRNA (Santa Cruz Biotech). The sgRNA sequences used for Gαi1 and Gαi3 KO are listed in **Table 1**.

**Gαi1/3 overexpression.** The recombinant adenovirus containing full-length Gαi1 (“Ad-Gαi1”, human or mouse) and Gαi3 (“Ad-Gαi3”, human or mouse) were described earlier 29. Virus was filtered, enriched and added to cultured MEFs, glioma cells or bmLCs. Stable cells were established following selection by puromycin, and Gαi1/3 overexpression confirmed by Western blotting.

**Glioma cell functional assays.** Cell growth, proliferation, CCK-8 viability and EdU staining assays 26, 34, 35 and cell migration by the “Transwell” assays 29, 36, 37, were carried out using previously described protocols.

**The orthotopic primary-derived xenograft assay**. For intracranial tumor implantation, primary human glioma cells or bmLCs (5 × 10^5^ cells of each mouse in 200 µL of Matrigel gel/10% FBS medium, with different genetic treatments) were implanted using the previously described coordinates 26, 38. Magnetic resonance imaging (MRI) was carried out to visualize tumor xenografts. On the day when the first mouse in any group exhibited symptoms (severe fever, vomiting, or greater than 15% body weight loss), all groups were sacrificed and tumors isolated through surgery. Tumor volumes were measured by the formula: π/6 × larger diameter × (smaller diameter)^2^. Immunohistochemistry (IHC) was performed using the previously described procedures 26. All animal procedures were approved by Soochow University Ethics Review Board.

**Statistical analysis.** The* in vitro* experiments were replicated three times or more, and data expressed as means ± standard deviation (SD). To examine statistical differences among different groups one-way ANOVA was carried out with multiple comparisons performed by post hoc Bonferroni test (SPSS 18.0). ***P*** values < 0.05 were considered statistically significant. A two-tailed unpaired t test (Excel 2007) was applied to examine significance between two treatment groups.

## Results

### Gαi1 and Gαi3 double knockout inhibits NLGN3-induced Akt, Erk and mTORC1 activation in mouse embryonic fibroblasts (MEFs)

**Figure 1:**
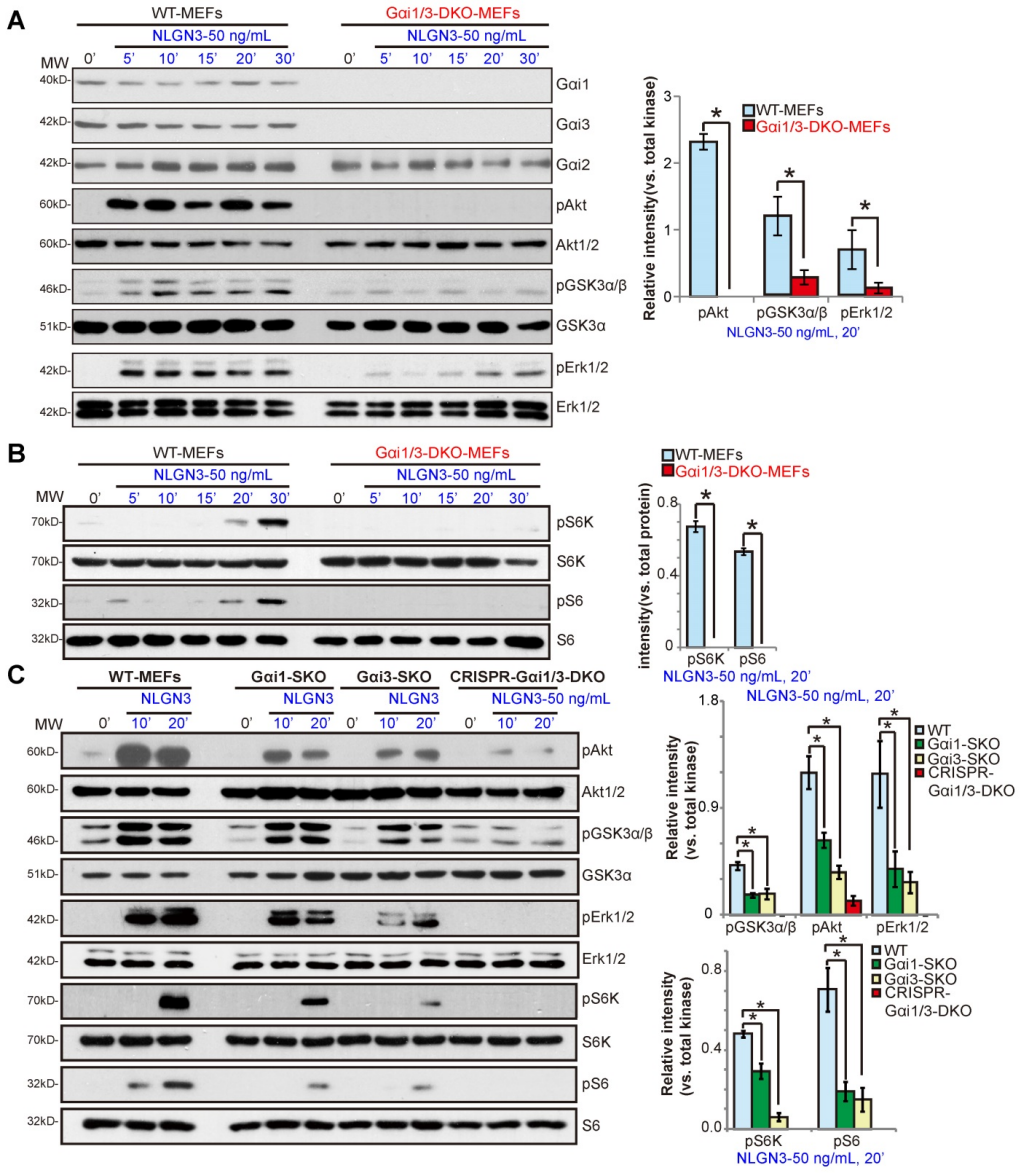
** Gαi1 and Gαi3 double knockout largely inhibits NLGN3-induced Akt, Erk and mTORC1 activation in MEFs.** Wild-type (WT), Gαi1 and Gαi3 double knockout (DKO) (**A**-**B**), Gαi1, or Gαi3 single knockout (SKO) (**C**) mouse embryonic fibroblasts (MEFs), or WT MEFs with Gαi1 plus Gαi3 CRISPR/Cas9 KO constructs (“CRISPR-Gαi1/3-DKO”, **C**), were treated with NLGN3 (50 ng/mL) for applied time, tested by Western blotting of listed proteins in total cell lysates. “MW” stands for molecular weight (Same for all Figures). Data were expressed as mean ± standard deviation (SD, same for all Figures). Quantifications were from five replicate blot data. *P < 0.05.

To test whether Gαi proteins are required for NLGN3-induced signal transduction, wild-type (WT) and Gαi1 and Gαi3 double knockout (Gαi1/3 DKO) MEFs 28, 29 were treated with NLGN3 (50 ng/mL) for 5-30 min. Western blotting results demonstrated that NLGN3 significantly increased phosphorylation of Akt (Ser-473) and GSK3α/β (Ser21/9) in WT MEFs, whereas phosphorylation was impaired in Gαi1/3 DKO MEFs (Figure **1A**). Furthermore, NLGN3-induced Erk activation, tested by p-Erk1/2 at Thr202/Tyr204, was also attenuated in the DKO MEFs (Figure **1A**). In WT MEFs NLGN3 activated mTORC1 signaling by inducing phosphorylation of p70S6K1(“S6K”, Thr389) and S6 (Ser-235/236), which was abolished in Gαi1/3 DKO MEFs (Figure **1B**). Levels of total Akt, Erk1/2, S6K and S6 were equivalent between WT and DKO MEFs (Figure **1A-B**). These results show that NLGN3-induced Akt, Erk and mTORC1 activation was significantly inhibited in Gαi1/3 DKO MEFs.

In Gαi1 or Gαi3 single knockout (SKO) MEFs, NLGN3-induced phosphorylation of Akt, Erk1/2, S6K and S6 was partially inhibited (***P*** < 0.05 *vs.* WT MEFs, Figure **1C**), indicating that both Gαi1 and Gαi3 can be utilized for NLGN3-induced signaling. To control for possible epigenetic modifications in the knockout MEFs, the CRISPR/Cas9 gene-editing method was applied to knockout both Gαi1 and Gαi3 in WT MEFs. As shown, NLGN3-induced Akt, Erk and mTORC1 activation was almost completely blocked in Gαi1/3 KO MEFS (“CRISPR-Gαi1/3-DKO”) (Figure **1C**).

### Gαi1 and Gαi3 are required for NLGN3-induced Akt, Erk and mTORC1 activation in MEFs

**Figure 2:**
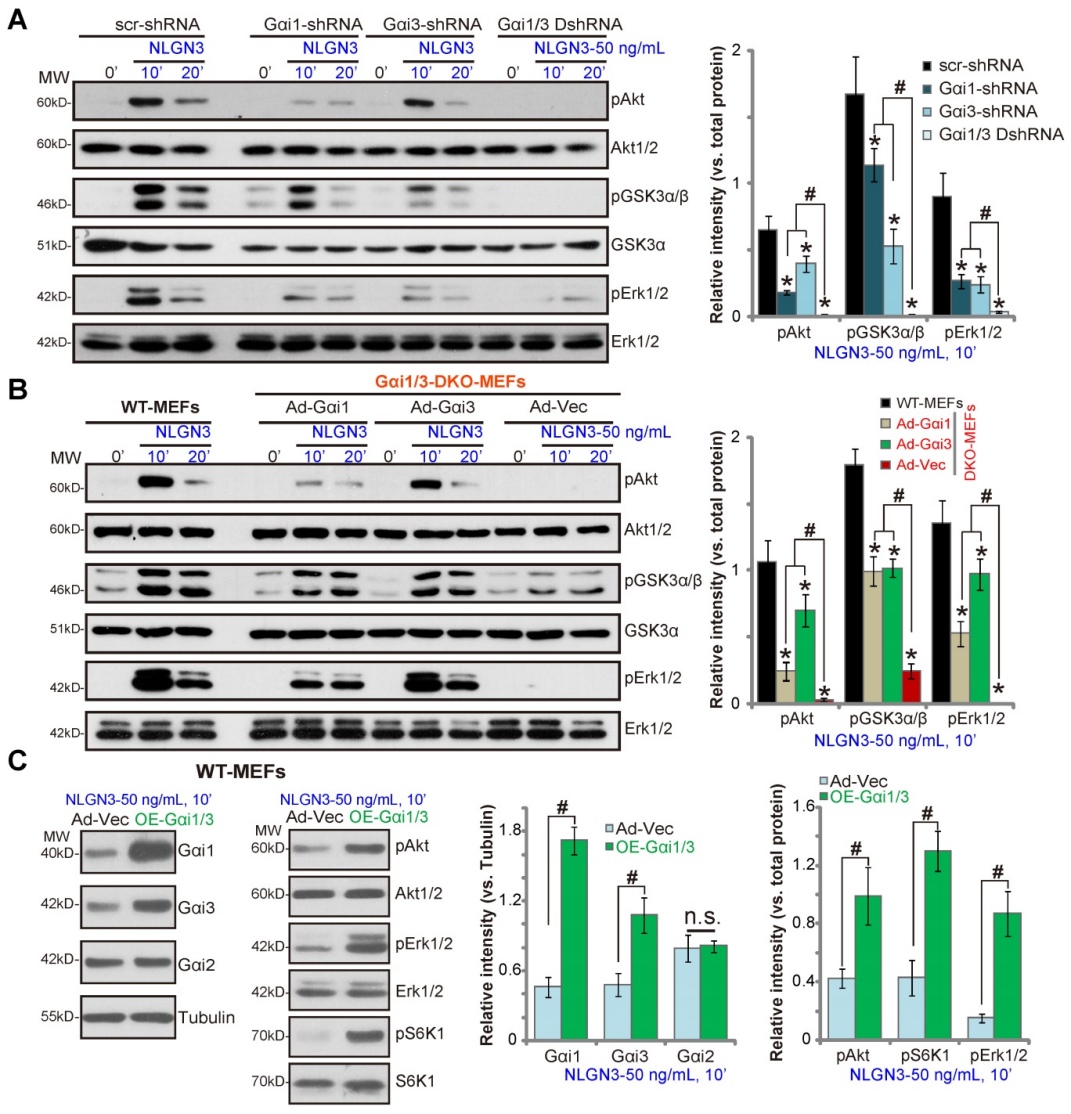
** Gαi1 and Gαi3 are required for NLGN3-induced Akt, Erk and mTORC1 activation in MEFs**. WT MEFs with the scramble control shRNA (“scr-shRNA”), the lentiviral Gαi1 shRNA, the lentiviral Gαi3 shRNA, or both (“Gαi1/3 DshRNA”), were treated with NLGN3 (50 ng/mL) for applied time periods, and tested by Western blotting of listed proteins in total cell lysates (**A**). Gαi1/3 DKO MEFs (**B**) or WT MEFs (**C**) were transfected with the adenovirus Gαi1 construct (“Ad-Gαi1”), the adenovirus Gαi3 construct (“Ad-Gαi3”) or the empty vector (“Ad-Vec”), treated with NLGN3 (50 ng/mL) for applied time periods, and tested of listed proteins. Quantifications were from five replicate blot data. *P < 0.05 vs. “scr-shRNA” (**A**) or WT MEFs (**B**). **^#^*P*** < 0.05. “n.s.” stands for ***P*** > 0.05 (no statistical differences, **C**).

To further confirm that Gαi1/3 are required for NLGN3-induced signaling the shRNA method was utilized. As described previously 29, Gαi1 shRNA lentiviral particles and/or Gαi3 shRNA lentiviral particles were added to WT MEFs to silence Gαi1 and/or Gαi3. As shown, shRNA-mediated single knockdown of Gαi1 or Gαi3 resulted in partial inhibition of Akt, GSK3α/β and Erk1/2 phosphorylation in response to NLGN3 (***P*** < 0.05 *vs.* MEFs with control shRNA, Figure **2A**). Significantly, Gαi1 and Gαi3 double knockdown by targeted shRNAs (“Gαi1/3 DshRNA”) led to further inhibition of NLGN3-induced signaling in MEFs (Figure **2A**).

To determine whether exogenously expressed Gαi proteins can rescue signaling, Gαi proteins were re-expressed in Gαi1/3 DKO MEF cells by infection with an adenovirus Gαi1 construct (“Ad-Gαi1”, no Tag) 28, 29 or adenovirus Gαi3 construct (“Ad-Gαi3”, no Tag) 28, 29. As shown, Ad-Gαi1 or Ad-Gαi3 restored NLGN3-induced Akt, GSK3α/β and Erk1/2 in DKO MEFs (Figure **2B**), further demonstrating that Gαi1 and Gαi3 are required for NLGN3-induced signaling. Conversely overexpression of Ad-Gαi1 (adenovirus-packed Gαi1) and Ad-Gαi3 in WT MEFs significantly increased NLGN3-induced signaling. Stable Gαi1 and Gαi3 overexpressing MEFs (“OE-Gαi1/3”) were established. Protein levels of Gαi1 and Gαi3 (but not Gαi2) were significantly increased in OE-Gαi1/3 MEFs (***P*** < 0.05 *vs.* vector control MEFs, Figure **2C**), where NLGN3-induced Akt, Erk1/2 and S6K phosphorylation were significantly increased (***P*** < 0.05 *vs.* vector control MEFs, Figure **2C**). Therefore, Gαi1/3 overexpression in WT MEFs enhanced NLGN3-induced downstream signaling activation.

### Gαi1 and Gαi3 are essential for NLGN3 signaling in glioma cells

**Figure 3:**
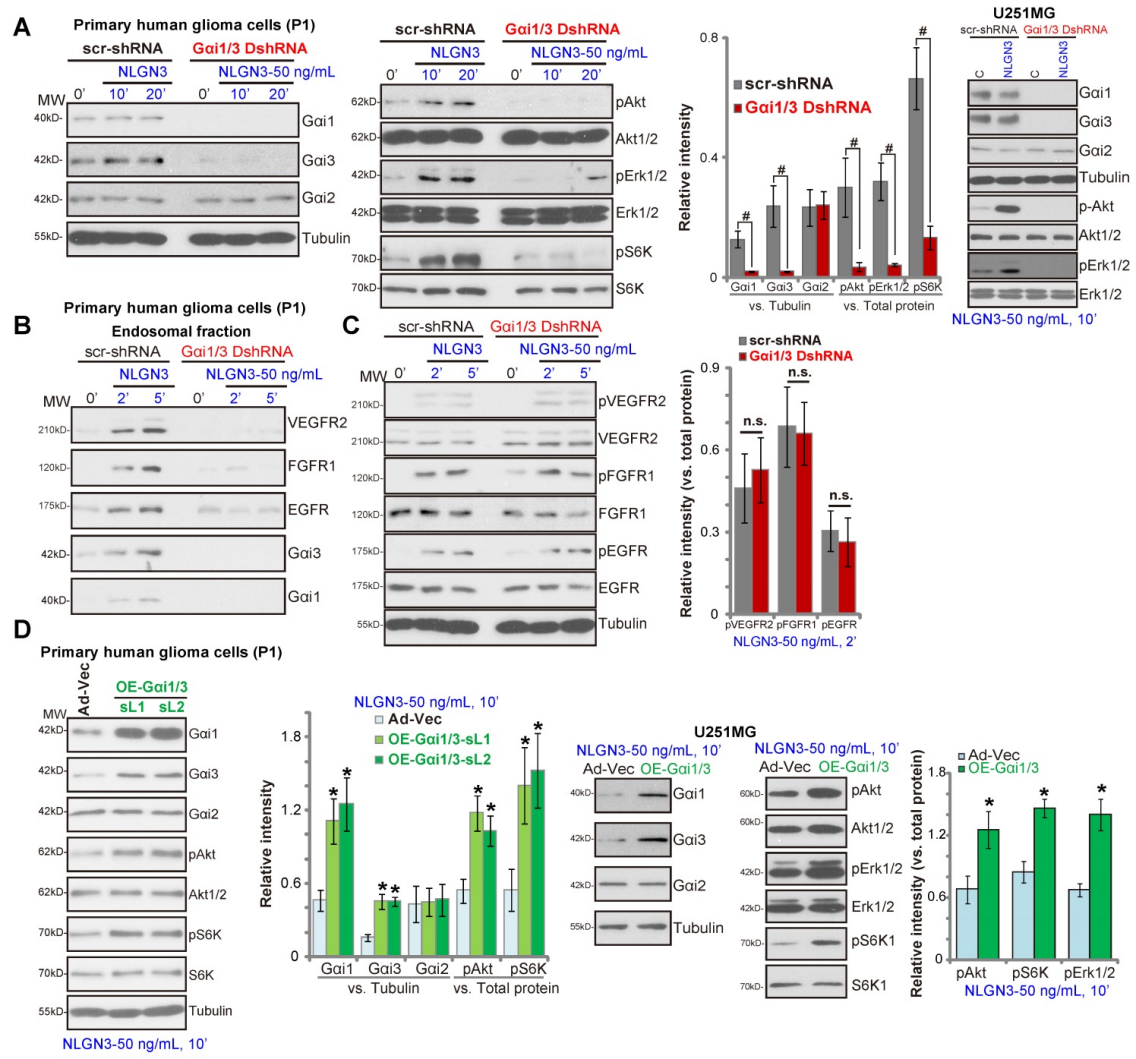
** Gαi1 and Gαi3 are essential for NLGN3 signaling in glioma cells.** The primary human glioma cells (“P1”) or U251MG cells, with Gαi1 shRNA plus Gαi3 shRNA (“Gαi1/3 DshRNA”) or the scramble control shRNA (“scr-shRNA”), were treated with NLGN3 (50 ng/mL) for applied time periods, listed proteins in total cell lysates or endosomal fractions were tested by Western blotting assays (**A**-**C**). P1 glioma cells or U251MG cells were transfected with the adenovirus Gαi1 construct plus the adenovirus Gαi3 construct (“OE-Gαi1/3”, two lines: “sL1/sL2”) or the empty vector (“Ad-Vec”), treated with NLGN3 (50 ng/mL) for 10 min, and tested by Western blotting assays of listed proteins (**D**). Quantifications were from five replicate blot data. **^#^*P*** < 0.05 (**A**).* *P* < 0.05 vs. “Ad-Vec” cells (**D**). “n.s.” stands for ***P*** > 0.05 (no statistical differences, **C**).

To silence Gαi1 and Gαi3 in glioma cells, primary human glioma cells (“P1”) were utilized. The pathology of P1 patients (female, 69) was: WHO grade IV, GFAP^+^, S100^+^, IDH1^—^, Nestin^+^, Olig2^+^, CD34^+^, Ki67^+^, p53-mutant, SOX10^+^, ATRX^+^, H3K27M^—^ and MGMT^—^. Cells were infected with Gαi1 shRNA lentivirus plus Gαi3 shRNA lentivirus 29. Following puromycin selection, stable glioma cells were established with depleted Gαi1 and Gαi3 (“Gαi1/3 DshRNA”, Figure **3A**). In control P1 glioma cells (expressing a scramble nonsense shRNA/“scr-shRNA”) NLGN3 potently induced phosphorylation of Akt, Erk1/2 and S6K, whereas signaling was almost blocked in P1 glioma cells transduced with Gαi1/3 DshRNA (Figure **3A**). In U251MG glioma cells, shRNA-mediated silencing of Gαi1/3 (“Gαi1/3 DshRNA”) similarly inhibited NLGN3-induced Akt and Erk1/2 phosphorylation (Figure **3A**).

Gαi1/3 association is essential for RTK endocytosis and endosomal translocation, a key step for downstream signal activation 28, 29. In response to NLGN3, multiple RTKs (VEGFR2, EGFR and FGFR1) as well as Gαi1 and Gαi3 were enriched in the endosomal fraction in P1 glioma cells (Figure **3B**), but not in Gαi1/3-silenced cells (Figure **3B**). RTKs expression and NLGN3-induced RTKs phosphorylation were not affected by Gαi1/3 silencing (Figure **3C**). These results indicated that Gαi1/3 are required for NLGN3-induced RTK endocytosis and downstream signaling transduction.

Conversely ectopic overexpression of Gαi1 and Gαi3 in P1 glioma cells significantly increased NLGN3-induced phosphorylation of Akt and S6K (Figure **3D**), without affecting their expression (Figure **3D**). For these experiments, Ad-Gαi1 (no tag) and Ad-Gαi3 (no tag) were transduced into P1 glioma cells, establishing Gαi1 plus Gαi3-overexpressed cells (“OE-Gαi1/3”, two stable cell lines, sL1/sL2) (Figure **3D**). Similarly, ectopic overexpression of Gαi1 and Gαi3 in U251MG cells (“OE-Gαi1/3”) enhanced NLGN3-induced Akt, S6K1 and Erk1/2 phosphorylation (Figure **3D**). Together, these results show that Gαi1 and Gαi3 are essential proteins for NLGN3 signaling in human glioma cells.

### Gab1 is a key adaptor protein for NLGN3 signaling in human glioma cells

**Figure 4:**
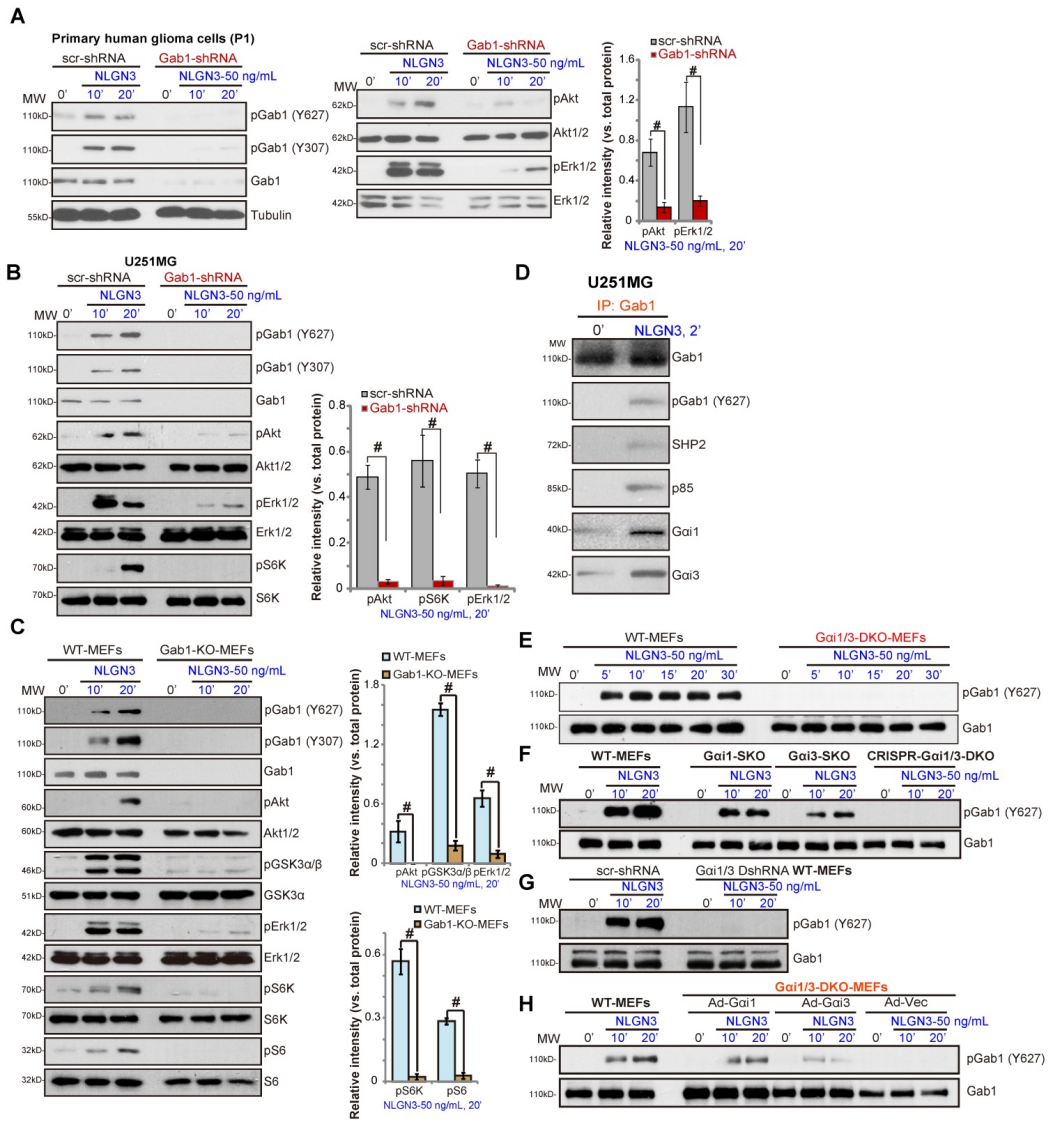
** Gab1 is a key adaptor protein of NLGN3 signaling.** The primary human glioma cells (“P1”) (**A**) or U251MG cells (**B**), with Gab1 shRNA or the scramble control shRNA (“scr-shRNA”), were treated with or without NLGN3 (50 ng/mL) for applied time periods, listed proteins in total cell lysates were tested by Western blotting. Wild-type (WT) and Gab1 knockout (Gab1-KO) MEFs were treated with NLGN3 (50 ng/mL) for applied time, tested by Western blotting of listed proteins in total cell lysates (**C**). U251MG cells were treated with NLGN3 (50 ng/mL) for 5 min, association of Gαi1/3-Gab1-SHP2-p85 was tested by co-IP assays (**D**). WT, Gαi1/3 DKO (**E**), Gαi1 or Gαi3 SKO (**F**), as well as WT MEFs with Gαi1 plus Gαi3 CRISPR/Cas9 DKO constructs (**F**) or Gαi1 shRNA plus Gαi3 shRNA (“Gαi1/3 DshRNA”) (**G**) were treated with NLGN3 (50 ng/mL) for applied time, total- and p-Gab1 were tested. Gαi1/3 DKO MEFs were transfected with the adenovirus Gαi1 construct (“Ad-Gαi1”), the adenovirus Gαi3 construct (“Ad-Gαi3”) or the empty vector (“Ad-Vec”), treated with NLGN3 (50 ng/mL) for applied time periods, total- and p-Gab1 were tested (**H**). Quantifications were from five replicate blot data. **^#^*P*** < 0.05.

Gab1 is a key Gαi1/3 adaptor protein required for RTK signal transduction 25, 27-29. We tested whether Gab1 is a required adaptor protein in NLGN3 signaling. NLGN3 was found to induce Gab1 activation, indicated by increased phosphorylation at Tyr-627 and Tyr-307 39 in P1 primary human glioma cells (Figure **4A**) and U251MG cells (Figure **4B**). Significantly, shRNA-mediated stable knockdown of Gab1 inhibited NLGN3-induced Akt, Erk1/2 and S6K phosphorylation in U251 and primary glioma cells (Figure **4A** and **B**).

In MEFs Gab1 KO abolished NLGN3 signaling by blocking downstream Akt-mTORC1 and Erk1/2 activation (Figure **4C**). Co-IP results in U251MG cells, Figure **4D**, demonstrated that, in response to NLGN3, Gab1 associated with Gαi1, Gαi3, SHP2 and p85. As Gab1-p85 association mediates PI3K-Akt activation and Gab1-SHP2 association is vital for Erk-MAPK cascade activation 39, we propose that Gab1 is a key adaptor protein for NLGN3 signaling in human glioma cells.

Importantly, NLGN3-induced Gab1 activation (p-Tyr at 627) was completely blocked in Gαi1/3 DKO MEFs (Figure **4E**) and in CRISPR-Cas9-induced Gαi1/3 DKO MEFs (Figure **4F**), being partially inhibited with Gαi1 or Gαi3 SKO (Figure **4F**). Additionally, shRNA-induced Gαi1/3 double knockdown also attenuated NLGN3-induced Gab1 activation in MEFs (Figure **4G**). Moreover, in DKO MEFs, re-expression of Gαi1 or Gαi3 restored NLGN3-induced Gab1 activation (Figure **4H**). These results demonstrate that Gab1, lying downstream of Gαi1/3, mediates NLGN3-induced Akt-mTORC1 and Erk activation.

### Gαi1 and Gαi3 mediates NLGN3-induced glioma cell progression *in vitro*

**Figure 5:**
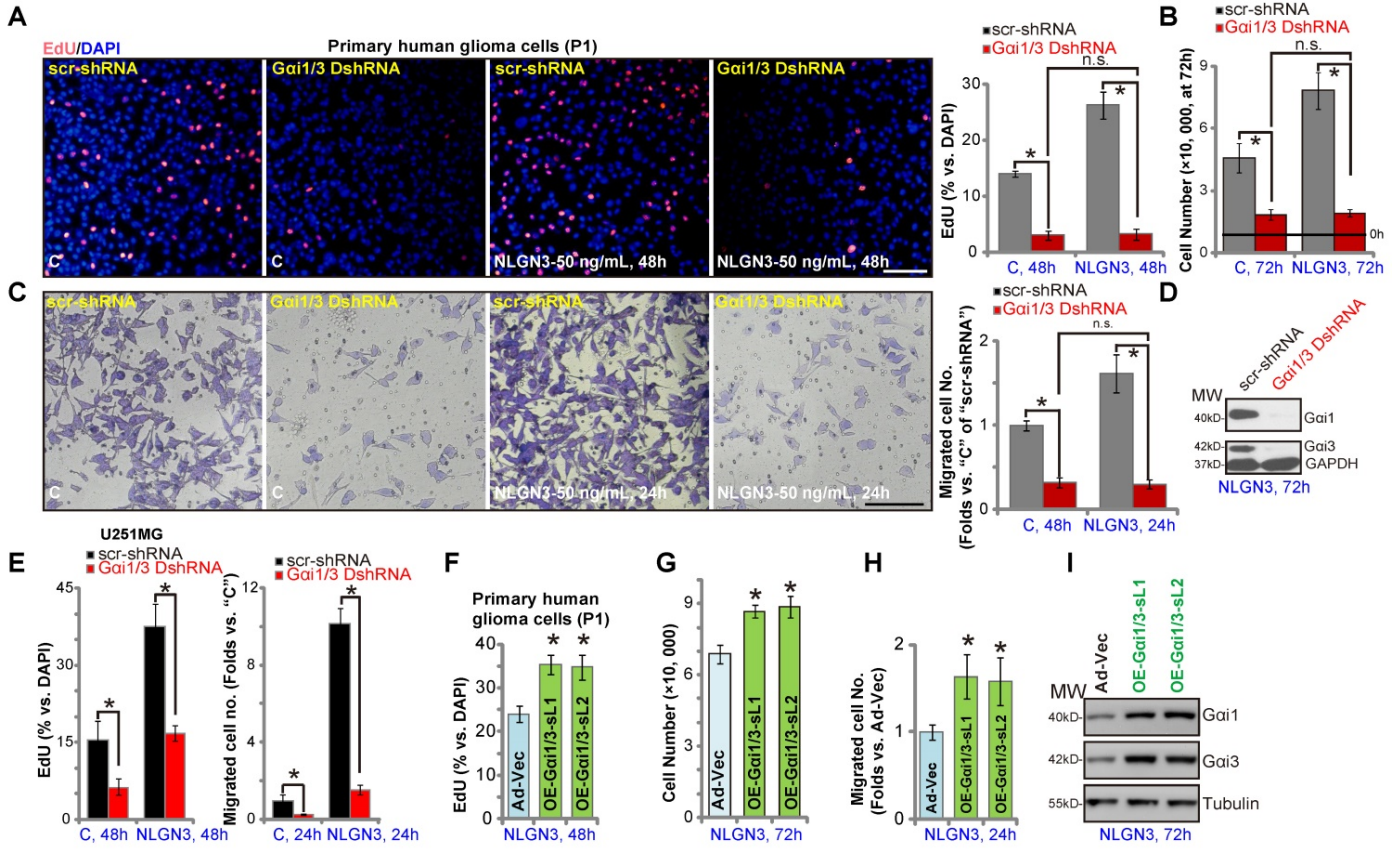
** Gαi1 and Gαi3 mediate NLGN3-induced glioma cell progression *in vitro*.** The P1 primary glioma cells or U251MG cells, with Gαi1 shRNA plus Gαi3 shRNA (“Gαi1/3 DshRNA”) or the scramble control shRNA (“scr-shRNA”), were treated with or without NLGN3 (50 ng/mL) for applied time periods, cell proliferation (**A** and **E**), growth (**B**), and migration (**C** and **E**) were examined by the assays mentioned in the text, with Gαi1 and Gαi3 expression tested as well (**D**, for P1 glioma cells). P1 glioma cells, with the adenovirus Gαi1 construct plus the adenovirus Gαi3 construct (“OE-Gαi1/3”, two lines: “sL1/sL2”) (**F**-**I**), were treated with or without NLGN3 (50 ng/mL) for applied time period, cell proliferation (**F**), growth (**G**), migration (**H**) and Gαi1/3 expression (**I**) were tested, with results quantified. For the EdU staining assay, ten random views were included to calculate EdU/DAPI ratios. For the “Transwell” assays, ten random views of each condition were included to calculate the average number of migrated cells. For all functional assays exact same number of viable cells of different genetic treatments were initially seeded into each well/dish (at 0h). “C” stands for medium control. Blotting data was repeated five times (**D** and **I**). *P < 0.05 (**A**-**E**). *P < 0.05 vs. “Ad-Vec” cells (**F**-**H**). “n.s.” stands for non-statistical difference (**A**-**C**). Scale bar=100 μm (**A** and **C**).

We next examined the role of Gαi1/3 proteins in glioma cell function. NLGN3 treatment promoted the growth of P1 primary glioma cells (with “scr-shRNA”), evidenced by increased EdU incorporation (Figure **5A**, at 48h) and cell number (Figure **5B**, at 72h). Significantly, NLGN3-induced effects were completely blocked by Gαi1 and Gαi3 shRNA (“Gαi1/3 DshRNA”) (Figure **5A** and **B**). Notably, basal glioma cell growth (by complete medium, no NLGN3) was also inhibited by Gαi1/3 DshRNA (Figure **5A** and **B**). Moreover, NLGN3 significantly induced glioma cell migration, evidenced by an increased number of migrated cells in “Transwell” tests (Figure **5C**), which was reversed by Gαi1/3 DshRNA (Figure **5C**). To confirm the efficiency of the applied shRNA, we showed that Gαi1 and Gαi3 proteins were silenced in Gαi1/3 DshRNA-expressing P1 glioma cells (with NLGN3 treatment, at 72h) (Figure **5D**). In U251MG cells, NLGN3-induced cell proliferation (EdU-positive nuclei ratio, Figure **5D**) and migration (“Transwell” cell number, Figure **5E**) were significantly inhibited by Gαi1/3 DshRNA, which also inhibited medium-induced basal cell proliferation and migration (Figure **5E**). These results demonstrated that Gαi1/3 silencing inhibited NGLN3-induced cell growth, proliferation and migration *in vitro*.

Conversely, ectopic overexpression of Gαi1 and Gαi3 (“OE-Gαi1/3”, see Figure **3**) in P1 glioma cells promoted NGLN3-induced cell proliferation (increased EdU incorporation, Figure **5F**), growth (Figure **5G**), and migration (“Transwell” assay, Figure **5H**). Western blotting results, Figure **5I**, confirmed Gαi1/3 overexpression in OE-Gαi1/3 glioma cells (72h after NLGN3 treatment). Notably, basal glioma cell proliferation (by complete medium, no NLGN3) and migration were enhanced with OE-Gαi1/3 (Figure **S1A**-**B**). Akt and Erk1/2 phosphorylation was augmented as well (Figure **S1C**).

### Gαi1 and Gαi3 are required for orthotopic growth of primary glioma xenografts in mouse brain

**Figure 6:**
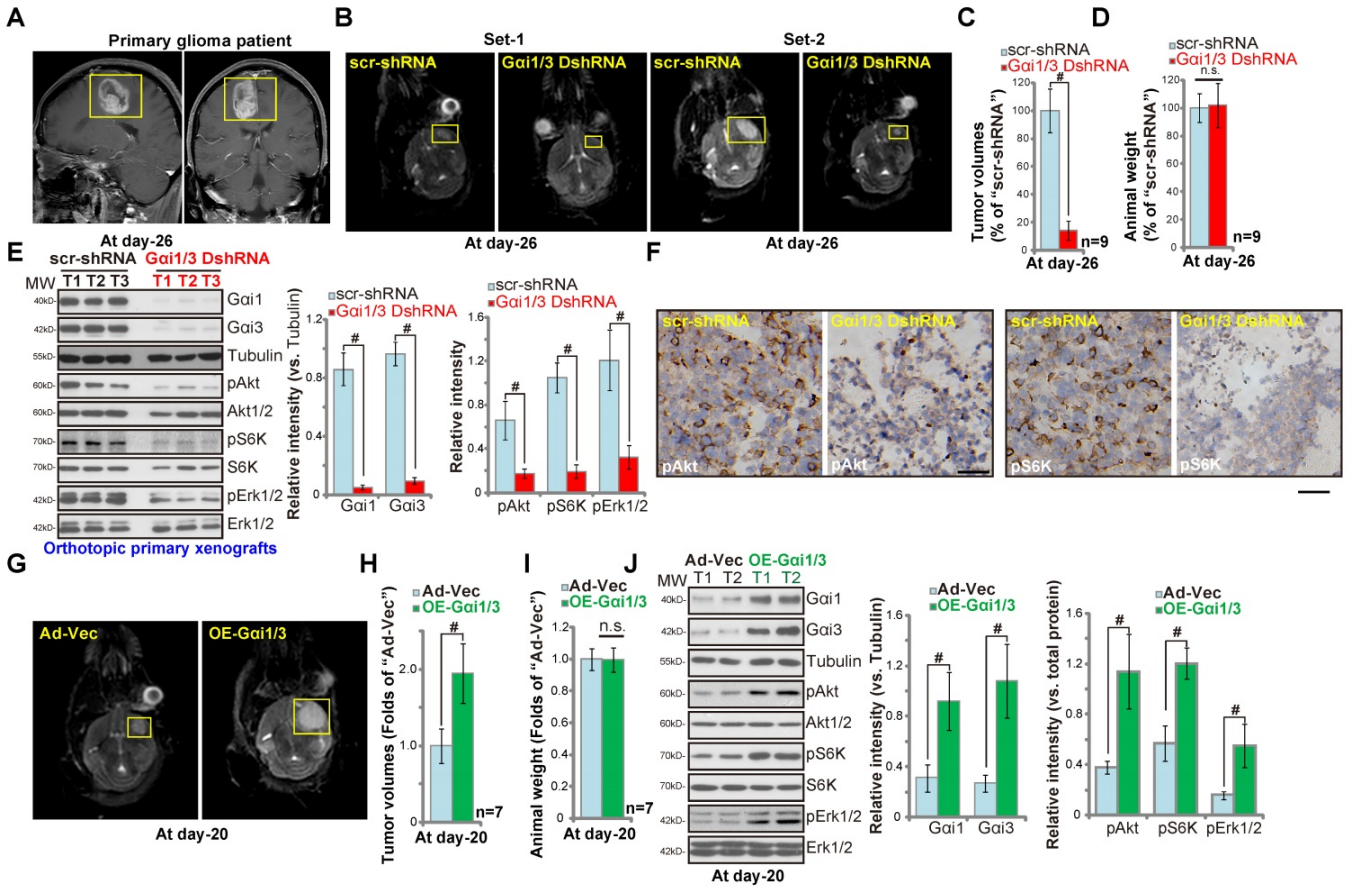
** Gαi1 and Gαi3 are required for orthotopic growth of primary glioma xenografts in mouse brain.** Brain MRI images of a primary glioma patient providing primary human glioma cells (“P1” cells, **A**). The exact same amount of glioma cells (5 × 10^5^ cells of each mouse), expressing Gαi1 shRNA plus Gαi3 shRNA (“Gαi1/3 DshRNA”), scramble non-sense shRNA (“scr-shRNA”), the adenovirus Gαi1 plus Gαi3 constructs (“OE-Gαi1/3”), or the empty vector (“Ad-Vec”), were intracranially injected to brains of nude mice (5-6 week old), after 26/20 days, representative MRI images of orthotopic glioma xenografts were presented (**B** and **G**); Animals were decapitated and tumors were isolated by surgery, tumor volumes (**C** and **H**) and mice body weights (**D** and **I**) were recorded. Tumor tissue lysates were tested by Western blot assay of listed proteins (**E** and **J**). Immunohistochemistry (IHC) images of p-Akt and p-S6K were shown (**F**). Western blotting quantifications were from five replicate blot data. IHC experiments were repeated in three pairs of tissues. **^#^*P*** < 0.05. “n.s.” stands for non-statistical difference (**D** and **I**). Scale bar=100 μm (**F**).

We performed orthotopic primary human glioma xenograft experiments using a previously-described protocol 26. P1 glioma cells, derived from a GBM patient (see MRI diagnosis in Figure **6A**), were intracranially (using the described parameters 38) injected into brains of nude mice. At experimental day-26, when the first mouse in the control group exhibited obvious symptoms, all the mice were sacrificed and tumors isolated 26. MRI imaging results confirmed that the growth of orthotopic primary glioma xenografts (Figure **6B**, yellow boxes) was significantly inhibited when expressing Gαi1 shRNA plus Gαi3 shRNA (“Gαi1/3 DshRNA”). Gαi1 and Gαi3 double silencing resulted in an 80% reduction of orthotopic glioma xenograft growth (Figure **6C**), and was significantly more effective than Gαi1 single silencing (50-60% inhibition, see our previous study 26). Mouse body weights were equivalent between the Gαi1/3 DshRNA and control shRNA groups (Figure **6D**). Gαi1/3 protein expression levels were significantly decreased in orthotopic primary xenografts with Gαi1/3 DshRNA, and levels of p-Akt, p-S6K and p-Erk1/2 inhibited (Figure **6E**). Immunohistochemistry (IHC) results, shown in Figure **6F**, further confirmed reductions in p-Akt and p-S6 in Gαi1/3-silenced orthotopic tumors (vs. control tumors). Therefore Gαi1/3 silencing significantly inhibited orthotopic growth of primary glioma xenografts.

In contrast, ectopic Gαi1/3 overexpression promoted orthotopic growth of primary glioma xenografts. Primary glioma cells transduced with Ad-Gαi1 plus Ad-Gαi3 (“OE-Gαi1/3”) or the vector control (“Ad-Vec”) were injected into the brains of the nude mice. At experimental day-20, when the first mouse in the OE-Gαi1/3 group exhibited obvious symptoms, all mice were sacrificed and the tumors isolated. MRI images showed that Gαi1/3-overexpressing primary glioma xenografts grew faster than the control tumors (with Ad-Vec) (Figure **6G**), presenting with higher tumor volumes (Figure **6H**). Mouse body weights were unchanged (Figure **6I**). Western blotting of tumor lysates confirmed Gαi1/3 overexpression in OE-Gαi1/3-tumor tissues, where levels of p-Akt, p-S6K and p-Erk1/2 increased (Figure **6J**). Taken together, Gαi1 and Gαi3 are required for orthotopic primary glioma xenograft growth in mouse brain.

### Orthotopic growth of brain-metastatic human lung cancer cells requires Gαi1 and Gαi3

**Figure 7:**
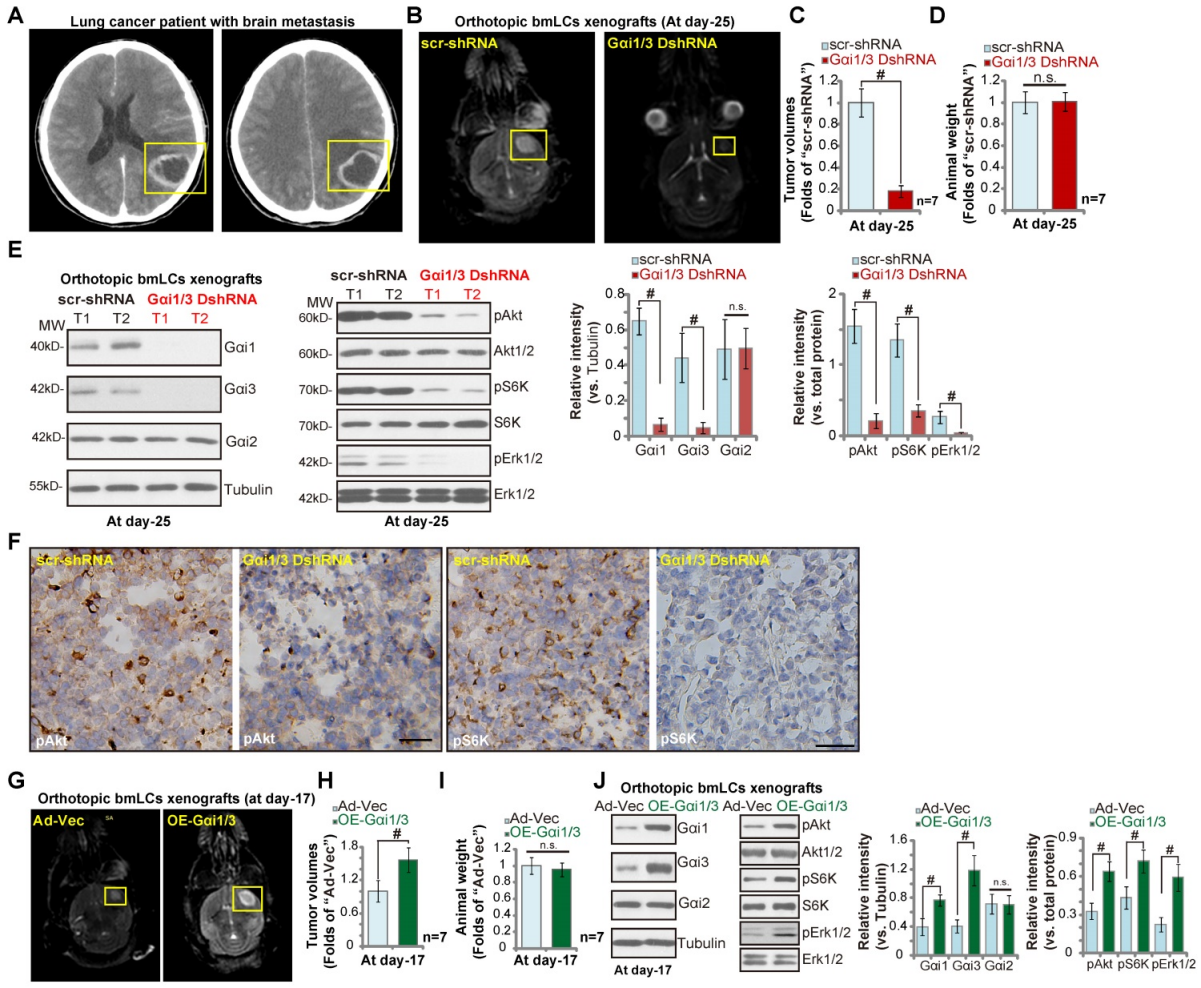
** Orthotopic growth of brain-metastatic human lung cancer cells requires Gαi1 and Gαi3.** Brain MRI images of a primary lung cancer patient with brain metastasis (**A**) providing the brain-metastatic human lung cancer cells (“bmLCs”). bmLCs (5 × 10^5^ cells of each mouse), expressing Gαi1 shRNA plus Gαi3 shRNA (“Gαi1/3 DshRNA”), scramble non-sense shRNA (“scr-shRNA”), the adenovirus Gαi1 plus Gαi3 constructs (“OE-Gαi1/3”), or the empty vector (“Ad-Vec”), were intracranially injected to brains of nude mice (5-6 week old); After 25/17 days, representative MRI images of orthotopic bmLCs xenografts were presented (**B** and **G**); Animals were decapitated and tumors were isolated by surgery, tumor volumes (**C** and **H**) and mice body weights (**D** and **I**) were recorded. The orthotopic bmLCs xenograft tissue lysates were tested by Western blot assay of listed proteins (**E** and **J**). Immunohistochemistry (IHC) images of p-Akt and p-S6K were shown (**F**). Western blotting quantifications were from five replicate blot data. IHC experiments were repeated in three pairs of tissues.**^#^*P*** < 0.05. “n.s.” stands for non-statistical difference (**D** and **I**). Scale bar=100 μm (**F**).

Next we tested whether Gαi1 and Gαi3 were also required for the growth of metastatic brain tumor. Brain-metastatic lung cancer cells, bmLCs, from a lung cancer patient with brain metastasis (see the MRI image of brain metastatic tumor, Figure **7A**) were obtained and primary-cultured. The bmLCs were infected with the lentivirus encoding Gαi1 shRNA plus Gαi3 shRNA (“Gαi1/3 DshRNA”) or scramble control shRNA (“scr-shRNA”), and stable bmLCs established after puromycin selection. In* ex vivo* cultured scr-shRNA control bmLCs, NLGN3 (50 ng, 10 min) increased phosphorylation of Akt, S6K and Erk1/2 (Figure **S2**). Gαi1 and Gαi3 double silencing by Gαi1/3 DshRNA potently inhibited NLGN3-induced signaling (Figure **S2**) in bmLCs.

Thereafter, cells were intracranially injected into brains of nude mice. At experimental day-25, when the first mouse in the control group exhibited obvious symptoms, all the mice were sacrificed. MRI image results confirmed that orthotopic bmLCs xenografts bearing Gαi1/3 DshRNA grew significantly slower than control bmLCs xenografts with scr-shRNA (Figure **7B**). Gαi1 and Gαi3 double shRNA led to over 80% reduction of orthotopic bmLCs xenograft growth (Figure **7C**). The mice body weights were indifferent between the two groups (Figure **7D**). Next, signaling proteins in orthotopic bmLCs xenografts were examined. Western blotting assay results, Figure **7E**, confirmed Gαi1 and Gαi3 silencing in bmLCs xenografts with Gαi1/3 DshRNA, while Gαi2 expression was unchanged (Figure **7E**). Importantly, phosphorylations of Akt, S6K and Erk1/2 were largely decreased in Gαi1/3 DshRNA-expressing orthotopic bmLCs xenografts (Figure **7E**). IHC images further confirmed decreased Akt-S6K phosphorylations in orthotopic bmLCs xenografts with Gαi1/3 DshRNA (Figure **7F**). These results showed that silencing of Gαi1 and Gαi3 inhibited orthotopic growth of bmLCs in mouse brain.

To further support out hypothesis, primary bmLCs, stably transduced with Ad-Gαi1 plus Ad-Gαi3 (“OE-Gαi1/3”), as well as the control cells with the vector control (“Ad-Vec”), were injected into the brains of the nude mice. At day-17, the first mice in the OE-Gαi1/3 group exhibited obvious neurological symptoms. MRI images showed that the orthotopic bmLCs xenografts with OE-Gαi1/3 were larger than the control xenografts (Figure **7G**). Tumor volumes of OE-Gαi1/3 bmLCs xenografts were significantly higher than those of Ad-Vec xenografts (Figure **7H**). The mice body weights were indifferent (Figure **7I**). Western blotting assaying of orthotopic bmLCs xenograft tissues confirmed Gαi1/3 overexpression as well as increased phosphorylations of Akt-S6K1 and Erk1/2 in OE-Gαi1/3 xenograft tissues (Figure **7J**). Together, these results show the orthotopic growth of bmLCs requires Gαi1 and Gαi3.

### Gαi3 upregulation in human glioma tissues

**Figure 8:**
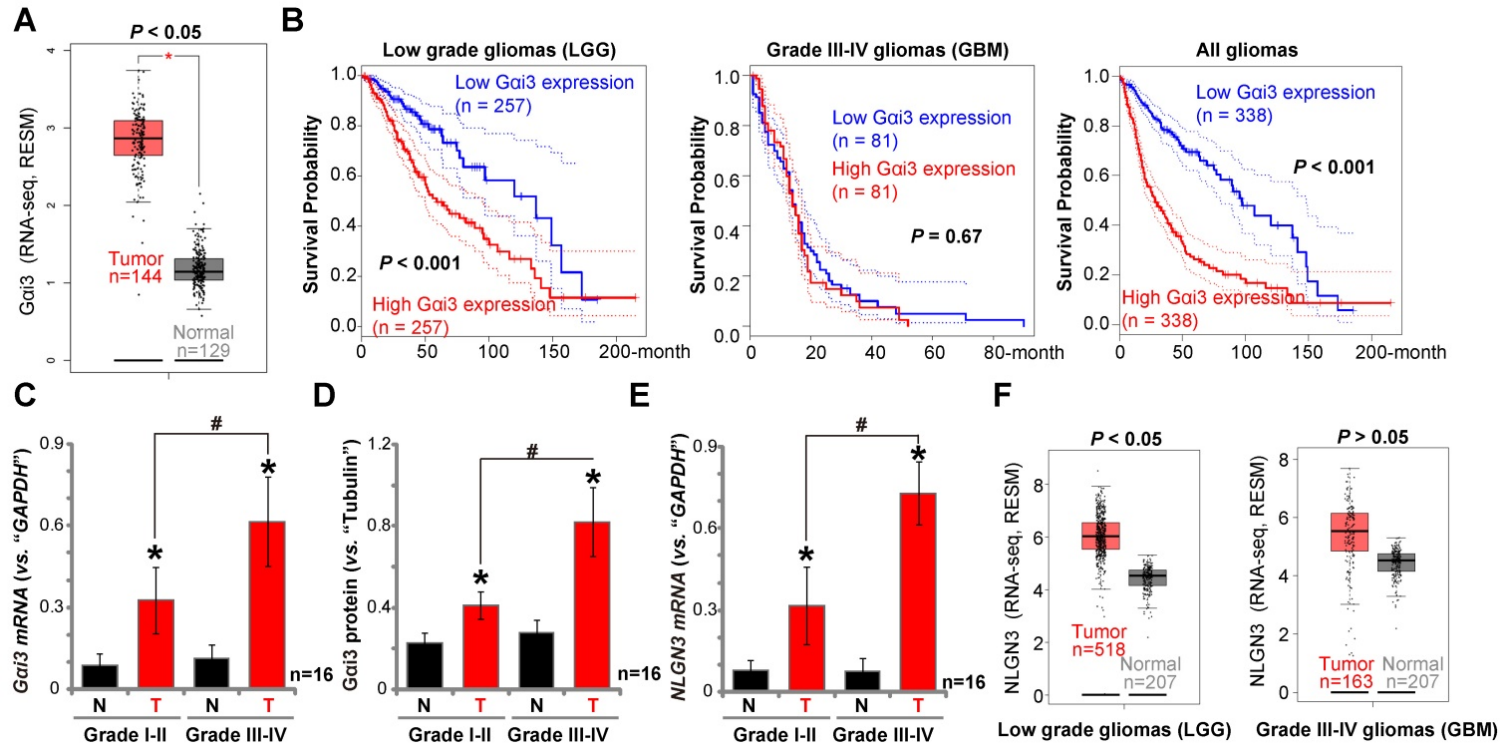
** Gαi3 upregulation in human glioma tissues.** TCGA database shows *Gαi3* and* NLGN3* expression (RNASeq, RSEM) in glioma tissues and normal brain tissues (**A** and **F**). Kaplan Meier Survival analyses of *Gαi3*-low and *Gαi3*-high glioma (LGG and GBM) patients (**B**). Human glioma tissues (“T”, 16 high-grade and 16 low-grade) and the paired normal brain tissues (“N”) were homogenized and dissolved in the tissue lysis buffer, *Gαi3 mRNA* and protein levels were tested by qPCR (**C**) and Western blotting (**D**), respectively, with results quantified. *NLGN3 mRNA* was tested by qPCR (**E**). * ***P*** < 0.05 *vs.*“N” group. **^#^*P*** < 0.05.

We have previously shown that *Gαi1 mRNA* and protein levels are upregulated in human glioma, correlating with high tumor grade 26. To examine Gαi3 expression in human glioma, we first consulted the TCGA database to examine RNA-Seq data. As shown, in human glioma tissues *Gαi3* mRNA expression is significantly upregulated [***P*** <0.05 vs. normal tissues] (Figure **8A**). The overall survival of the low grade glioma (LGG) patients with *Gαi3*-high gliomas is significantly lower than those with *Gαi3*-high gliomas (***P*** < 0.001, Figure **8B**). There is however no significant difference in the overall survival of *Gαi3*-low GBM patients and *Gαi3*-high GBM patients (Figure **8B**). This could be due to the extremely low overall survival of GBM patients (Figure **8B**). When combining all glioma data, we showed that the average survival of *Gαi3*-high glioma patients is significantly lower than those with *Gαi3*-low gliomas (***P*** < 0.001, Figure **8B**). These results indicate that *Gαi3* upregulation in human glioma is correlated with a poor overall survival.

To confirm the significance of the bioinformatics observations, we examined Gαi3 expression in human glioma specimens (“T”) and surrounding normal brain (“N”) tissues, from sixteen (16) low-grade (Grade I-II) tumors and another sixteen high-grade (Grade III-IV) tumors 26. qPCR results, Figure **8C**, demonstrated that *Gαi3* mRNA levels are significantly upregulated in human glioma tissue (*vs.* normal brain tissue, Figure **8C**). In late-stage gliomas (Grade III-IV, n=16) *Gαi3* mRNA is more highly upregulated than in early-stage gliomas (Grade I-II, n=16) (Figure **8C**). In the late-stage gliomas (Grade III-IV, n=16) *Gαi3 mRNA* is highly upregulated than that in early-stage gliomas (Grade I-II, n=16) (Figure **8C**). Western blotting quantification results confirmed Gαi3 protein upregulation in human glioma tissues (Figure **8D**), being more significant in the high-grade tumors (Figure **8D**).

In line with previous studies 18, 19, 40, *NLGN3 mRNA* levels were increased in human glioma tissues (Figure **8E**), and levels were higher in the late-stage gliomas (Figure **8E**). TCGA cohorts showed that NLGN3 transcripts are higher in glioma tissues when compared to normal brain tissues (Figure **8F**), being significant in LGG tissues (Figure **8F**). Therefore, Gαi3, similar to Gαi1 26, is upregulated in human glioma tissues, correlating with poor survival, high tumor grade and NLGN3 upregulation.

## Discussion

GPCR components, including GPR56 41, GPR55 42 and many others, are dysregulated in human glioma and in other brain tumors. cAMP contents and the adenyl cyclase (AC) activity were decreased in brain tumors 43. He *et al.*, showed that Gαs subunit was downregulated in medulloblastoma, functioning as a tumor suppressor by blocking Sonic hedgehog signaling 44. Lelievre *et al.,* indicated that AC-mediated cAMP regulation plays an important cooperating role in the genesis of medulloblastoma 45. Importantly, Gαi-coupled GPCRs, including cannabinoid receptors 46, CXCR4 47, 48, dopamine D2 receptors 24 and melatonin receptor II (MTII) 49, were reported to be important for glioma progression. Pharmacological inhibition or genetic silencing of the Gαi-coupled GPCRs has potent anti-tumor effects 46-49. Similarly, Urotensin II-activated Urotensin II receptor coupled to both Gαi/o and Gα13 to activate PI3K-PIP3-GEF-Rac-Cdc42 signaling cascade 50. Le Joncour *et al.,* reported that Urotensin II and its receptor were overexpressed in human glioma, important for angiogenesis of glioma 51. Consistently, CXCR4-mediated Gαi activation and cAMP suppression is stimulated by the Sonic hedgehog pathway 52.

Our results show that Gαi1 and Gαi3 are essential players in NLGN3 signaling. In MEFs, Gαi1/3 DKO blocked NLGN3-induced Akt-mTORC1 and Erk-MAPK activation, with Gαi1 or Gαi3 SKO resulting in partial inhibition. Furthermore, in WT MEFs shRNA-mediated silencing of Gαi1 and Gαi3 attenuated Akt-mTORC1 and Erk-MAPK activation by NLGN3. Ectopic Gαi1/3 expression restored NLGN3-induced Akt-mTORC1 and Erk-MAPK activation in Gαi1/3 DKO MEFs, and enhanced NLGN3-induced signaling in WT MEFs. Similarly in established (U251MG) or primary human glioma cells, NLGN3-induced Akt-mTORC1 and Erk-MAPK activation was inhibited by Gαi1/3 shRNA, but enhanced with ectopic Gαi1/3 overexpression. These results show that Gαi1 and Gαi3 are required for NLGN3-induced mitogenic signal transduction.

Sustained and constitutive activation of multiple key RTKs is a primary driving force for glioma cell progression 53, 54, causing hyperactivation of downstream mitogenic pathways, including PI3K-Akt-mTOR and Erk-MAPK 54, 55. There are reports of GPCR-RTKs crosstalk in glioma cells. For example, Huang *et al.,* found that activation of the formylpeptide receptor induced trans-activation of EGFR via Gαi proteins, exacerbating malignant behaviors in glioblastoma cells 56. Ghosh *et al.,* found that Gαi-GIV (a novel non-receptor GEF for Gαi 57) complex directly associated with and activated EGFR to promote cancer cell migration and proliferation 57.

Studies have shown that NLGN3 induces phosphorylations of multiple RTKs as well as downstream PI3K-Akt-mTOR and Erk-MAPK cascades, responsible for glioma cell growth and proliferation 19. We found that Gαi1/3 proteins associate with activated RTKs (VEGFR and TrkB (28, 29) and are required for their endocytosis and downstream signaling. Here in glioma cells, Gαi1/3 silencing, by targeted shRNAs, blocked NLGN3-induced RTKs endosomal translocation and subsequent Gab1 activation, without affecting RTK phosphorylation and expression. Importantly, Gab1 silencing inhibited NLGN3-induced downstream signaling activation in glioma cells. These results suggest that Gαi1 and Gαi3 mediate NLGN3-induced signal transduction by promoting RTK endocytosis and adaptor protein Gab1 activation.

Functional studies demonstrate that Gαi1/3 are essential in mediating NLGN3-induced glioma cell progression *in vitro* and orthotopic primary glioma xenograft growth *in vivo*.NLGN3-stimulated glioma cell growth, proliferation and migration were largely inhibited by Gαi1/3 silencing, but enhanced by ectopic Gαi1/3 overexpression. Glioma xenografts are unable to grow in NLGN3 KO brain suggesting an essential dependency on NLGN3 for orthotopic glioma growth 28, 29. Significantly, our results show that the growth of orthotopic primary glioma xenografts was largely inhibited with Gαi1/3 silencing. Conversely the Gαi1/3-overexpressed primary glioma xenografts grew significantly faster than the control tumors. We concluded that possibly by mediating NLGN3-induced signaling transduction, Gαi1/3 are essential for glioma cell progression *in vitro* and *in vivo*.

One important finding of this study is that Gαi1 and Gαi3 are required for the orthotopic growth of bmLCs in mouse brain. One possibility is that neuron-secreted NLGN3 is also important for orthotopic growth of bmLCs. Indeed, *ex vivo* cultured bmLCs were responsible to NLGN3. NLGN3 activated Akt-mTOR and Erk1/2 signalings, and promoted bmLCs proliferation and migration *in vitro*. Such actions were almost abolished with Gαi1/3 DshRNA. Importantly, orthotopic growth of bmLCs in mouse brain was largely inhibited with Gαi1/3 DshRNA, but augmented with ectopic Gαi1/3 overexpression. In addition, Akt-mTOR and Erk1/2 activation in orthotopic bmLCs xenograft tissues was inhibited with Gαi1/3 DshRNA, but enhanced with Gαi1/3 overexpression. Other mitogens, including the BDNF and VEGF, could also be important contributors for the growth of brain-metastatic cancer cells 18. Interestingly, we have previously shown that BDNF- and VEGF-induced mitogenic signalings rely on Gαi1/3 28, 29. This could also demonstrate the essential role of Gαi1/3 in mediating orthotopic growth of bmLCs in mouse brain.

NLGN3 is a crucial factor for neuron-glioma synapse formation and neuronal activity-dependent glioma progression 14, 15, 20. Interestingly, Hanahan and his colleagues have shown that brain metastatic breast-cancer cells can form a specialized type of pseudo-tripartite synapse with glutamatergic neurons 58. This will allow cancer cells to take up glutamate and activate GluN2B-mediated NMDAR (N-methyl-D-aspartate receptor) signaling, required for metastatic cancer cell growth 58. Thus, another possibility is that bmLCs might form synapses with neurons to activate glutamate signaling, and neuron-secreted NLGN3 could be essential for the process. The underlying mechanisms warrant further characterizations.

NLGN3 expression levels negatively correlate with overall survival of glioblastoma patients 18, 19. Furthermore high NLGN3 expression is associated with glioma recurrence 40. Our current and previous results 26 have demonstrated that Gαi1 and Gαi3 levels are significantly upregulated in human glioma tissues, and their upregulation is correlated with poor patients survival, high tumor grade and NLGN3 upregulation. Gliomas, especially the high grade GBM, are composed of a pathologically heterogeneous mixture of cells exhibiting different cellular and nuclear polymorphism 59-61. Therefore, Gαi1/3 and NLGN3 upregulation in glioma tissues might be derived from both glioma cells and possible other cells in the tumor bulk (immune cells, endothelial cells and cancer stem cells, *etc*). Although the underlying mechanisms of Gαi1/3-mediated NLGN3 signaling and glioma cell progression need further characterization, the results of the present and previous studies 26 strongly indicate that Gαi1 and Gαi3 could be valuable therapeutic targets for treating human glioma.

## Conclusions

Gαi1/3 mediation of NLGN3-induced signaling is essential for glioma growth *in vitro* and *in vivo*.

## Figures and Tables

**Table 1:** Primers and sgRNA sequences utilized in the study.

Genes	qPCR primers
*GAPDH* Forward	5'-GTCGTGTGAACGGATTTG-3'
*GAPDH* Reverse	5'-AAGATGGTGATGGGCTTCC-3'
*NLGN3* Forward	5'- GTCTGGTTCACTGCCAACTTGG -3'
*NLGN3* Reverse	5'- CCGTCATTATCCGCTAAGTCCTC-3'
*GNAI3* Forward	5'- CACTTCACCTGTGCCACAGACA-3'
*GNAI3* Reverse	5'- GTCTGGTCTCAACACTCCACAC-3'
*sgRNA*	Target DNA Sequence
*Murine GNAI3 sgRNA*	GGCTCGTATGATTGCAATGA
*Murine GNAI1 sgRNA*	CCATCATTAGAGCCATGGGG
