# Supplementary material for: Neuronal-driven glioma growth requires Gαi1 and Gαi3
Source: Theranostics. 2021 Jul 25;11(17):8535–49. doi: 10.7150/thno.61452 (PMC8343996; doi:10.7150/thno.61452)
Supplement: Supplementary file 1 — Supplementary figures. [file thnov11p8535s1.pdf]

Figure S1

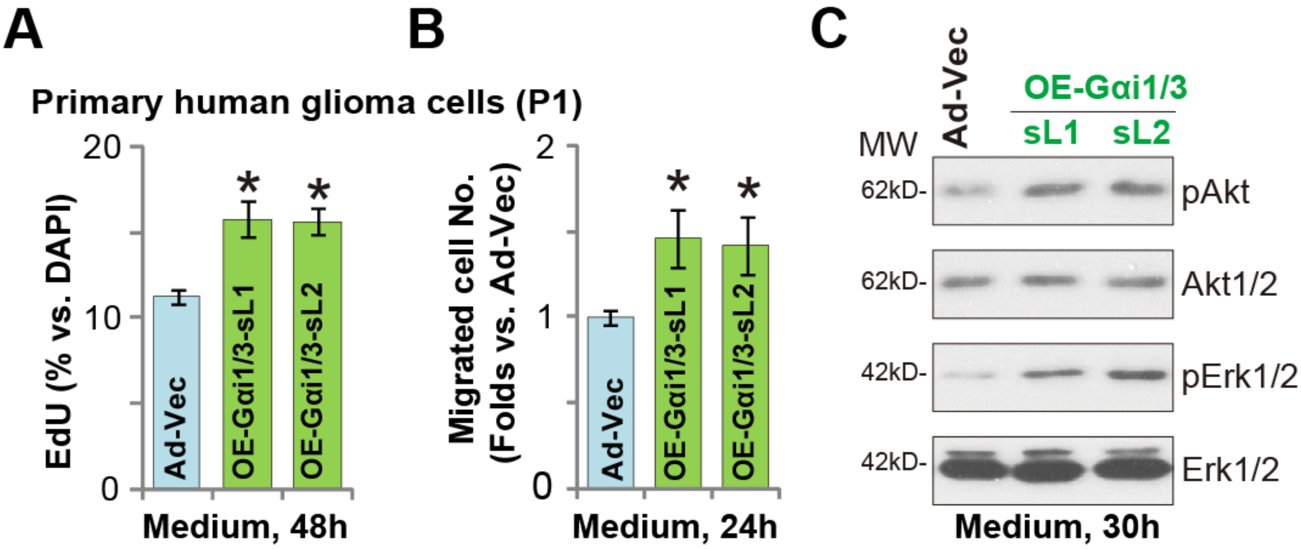

Figure S1. P1 glioma cells, with the adenovirus Gαi1 construct plus the adenovirus Gαi3 construct ("OE-Gαi1/3", two lines: "sL1/sL2") or empty vector ("Ad-Vec"), were cultured in complete medium for applied time period, cell proliferation (**A**, by recording EdU-positive nuclei ratio) and migration (**B**, "Transwell" assays) were tested; Listed proteins were shown (**C**). Blotting data was repeated five times (**C**). *$P < 0.05$ vs. "Ad-Vec" cells.

Figure S2

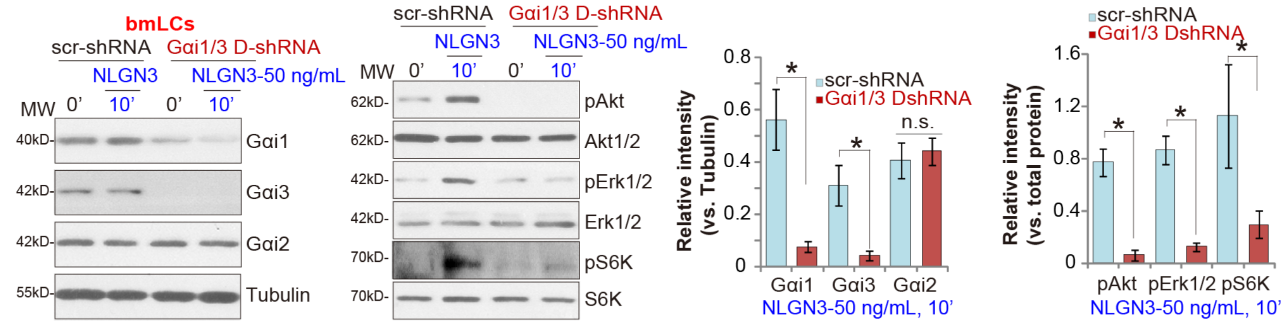

Figure S2. The brain-metastatic human lung cancer cells ("bmLCs"), with Gαi1 shRNA plus Gαi3 shRNA ("Gαi1/3 DshRNA") or the scramble control shRNA ("scr-shRNA"), were treated with NLGN3 (50 ng/mL) for applied time periods, expressions of the listed proteins were tested by Western blotting assays; Quantifications were from five replicate blot data. * $P < 0.05$. "n.s." stands for no statistical differences.
